# Three-dimensional network of nitrogen-doped carbon matrix-encapsulated Si nanoparticles/carbon nanofibers hybrids for lithium-ion battery anodes with excellent capability

**DOI:** 10.1038/s41598-022-20026-9

**Published:** 2022-09-26

**Authors:** Ruye Cong, Minsang Jo, Angelica Martino, Hyun-Ho Park, Hochun Lee, Chang-Seop Lee

**Affiliations:** 1 Department of Chemistry, Keimyung University, Daegu, 42601 Korea; 2 Department of Energy Science and Engineering, DGIST, Daegu, 42988 Korea

**Keywords:** Chemistry, Materials science

## Abstract

Three-dimensionally structured silicon (Si)–carbon (C) nanocomposites have great potential as anodes in lithium-ion batteries (LIBs). Here, we report a Nitrogen-doped graphene/carbon-encapsulated Si nanoparticle/carbon nanofiber composite (NG/C@Si/CNF) prepared by methods of surface modification, electrostatic self-assembly, cross-linking with heat treatment, and further carbonization as a potential high-performance anode for LIBs. The N-doped C matrix wrapped around Si nanoparticles improved the electrical conductivity of the composites and buffered the volume change of Si nanoparticles during lithiation/delithiation. Uniformly dispersed CNF in composites acted as conductive networks for the fast transport of ions and electrons. The entire tightly connected organic material of NG/C@Si and CNF prevented the crushing and shedding of particles and maintained the integrity of the electrode structure. The NG/C@Si/CNF composite exhibited better rate capability and cycling performance compared with the other electrode materials. After 100 cycles, the electrode maintained a high reversible specific capacity of 1371.4 mAh/g.

## Introduction

Environmental problems caused by global warming have been bringing great threats to our survival. The most important factor leading to global warming is the massive emissions of pollutants such as carbon dioxide. The main sources of carbon (C) emissions are the burning and transportation of fossil fuels. Therefore, the sustainable development of new energy technologies are urgent research topics^1–3^. Rechargeable lithium (Li)-ion batteries (LIBs) are promising candidates for energy storage applications in electric/hybrid vehicles and portable electronic devices, because of their high-energy density, wide working voltage, low self-discharge, large output power, high storage capacity, good cycle performance, and environmental compatibility. To meet the growing demand for energy storage equipment, the development of LIBs with better energy density and cycle performance has become more important^4–6^. Among various anode materials, silicon (Si) is one of the most promising materials due to its high theoretical specific capacity (~ 4200 mAh g^−1^), low working potential (~ 0.4 V vs. Li/Li^+^), abundance, low price, and environmental safety. Si has become a substitute for traditional graphite-based anode materials with theoretical capacity of 372 mAh g^−1^^7–9^. However, the practical application of Si-based materials in commercial LIBs faces several challenges. The low conductivity of Si-based materials results in poor electrode rate performance. The volume change (~ 300%) of the Si particles during a cycle causes the electrode material to smash, fall off, and lose electronic contact with a current collector. It causes rapid decay in battery capacity, shortening of cycle life, and damage of battery cells. Finally, a typical electrolyte forms a solid electrolyte interface (SEI) on the Si surface at a potential of < 1 V. During a volume change, the SEI may crack and expose Si particles; thus, more SEI is formed on the exposed Si surface. The SEI film increases continuously the total layer thickness of Si particles and fills up quickly the electrode holes, preventing the transmission of Li-ions and electrons. This result in an increase and decrease in impedance and conductivity, respectively, which affect the cycle stability of a battery^10–13^.

To solve the above-mentioned problems, Si nanoparticles have been coated/encapsulated by C-based materials (e.g., amorphous C from various C precursors, graphene (G), C nanotubes, and carbon nanofibers (CNF) with high graphitization strength)^14–16^. Si nanoparticles can shorten the distance of Li^+^ transmission path and maintain the volume change during a cycle. The inert/active matrix can act as a buffer layer with high conductivity and strong mechanical strength, which enhance structural stability and conductivity. Recently, G has been recognized as a high-efficiency coating material in the preparation of LIBs due to its unique properties such as high electrical conductivity, chemical stability, high thermal stability, excellent mechanical flexibility, and high theoretical surface area. It has potential application in energy storage. G-based Si/C composite materials can alleviate the volume change of Si nanoparticles and form a stable SEI film. It can also improve the electrical conductivity and Li storage performance of Si nanoparticles. In addition, the voids created by vacancy defects in G open up channels for ion transmission, increase the permeability of G to ions, and improve ion diffusion coefficient and reactivity. Doping defects and vacancies enhance the interaction between adsorbed atoms and G^17,18^. Graphene oxide (GO) is the most common G precursor used in the synthesis of G nanocomposites. Nitrogen (N) atom-doped reduced GO (N-doped rGO) can improve effectively the physical and electrochemical properties of G. Roshni Yadav et al. reviewed the synthesis, characterization, and potential applications of N-doped G^19^. When a N atom is doped into G, three common bonding configurations in the C lattice including quaternary N (or graphite N), pyridine N, and pyrrole N are observed. Generally, pyridine N bonds with two C atoms at the edges or defects of graphene and contributes a p electron to the π system. Pyrrole N means that the N atom contributes two p electrons to a π system and is bonded unnecessarily to a five-membered ring (e.g., pyrrole). The quaternary N is the N atom that replaces the C atom in the hexagonal ring. Pyridine N and quaternary N are sp^2^ hybridized while pyrrole N is sp^3^ hybridized. Graphite N, pyridine N, and pyrrole N improve the conductivity of a material, determine the electrochemical activity, and improve the charge transfer, respectively^20–23^. Studies have shown that N atoms with two lone pair electrons are more electronegative than C atoms. Therefore, the electron density of N-doped C becomes lower with stronger electrochemical activity. Due to the electronegatively of N, N lone pair electrons are hybridized with the G π system. In a graphite plane, a *p*–π conjugate is formed between the lone pair of N electrons and the π electrons of G, which improves the charge transfer ability of N-doped G; thereby, increasing the conductivity^24–26^.

Significant progress has been made in the Si/N-doped rGO composites that improve battery performance; however, their practical application still faces challenges. It has been found that due to the strong van der Waals interaction, G is prone to form irreversible particle agglomeration, which hinders the effective charge transfer during a cycle, resulting in poor conductivity of an electrode material. In addition, the dispersibility of Si nanoparticles in an aqueous system is poor. The superpositions among particles increase the contact resistance and reduce the electrochemical performance of the active material^27^. According to previous reports, Yinjie Cen et al. studied the effect of surface treatment on the electrochemical performance of silicon/graphene nanocomposites. They found that after the introduction of silane reagent into Si nanoparticles pretreated with piranha solution, stronger bonding with GO resulted in a more stable composite structure^28^. Ren Na et al. proposed that the thickness of a Si modified layer is controlled by the hydrolysis time of aminopropyltriethoxysilane (APTES)^13^. However, the simple combination of Si nanoparticles and G sheets reduces the contact area because of the single point-to-point contact mode between Si and G. This contact mode leads to an increase in the diffusion distance of Li^+^ through the G layers, affecting the transmission of electrons and ions^29^. Moreover, due to the different volume expansion rates between Si and G, Si nanoparticles are more likely to peel off from G after several charge/discharge cycles, resulting in a decrease in cycle performance. Meanwhile, CNF has high conductivity, good mechanical strength, high specific surface area, and chemical stability. CNF interspersed between Si/G composites forms a robust three-dimensional (3D) structure, which can increase effectively the specific surface area to provide an open channel for electrolyte immersion, reduce the stacking of G layers, and shorten the transmission distance of electrons and Li^+^. It can also accommodate and buffer the volume change of Si, prevent the electrode structure from cracking, and prevent Si nanoparticles from falling off the C base due to the surface area expansion; thus, the electrode shows good electrical conductivity and stable mechanical properties^30^.

Here, we report citric acid as a low-cost, environmentally friendly, non-toxic cross-linking agent, GO as C source, and melamine formaldehyde resin (MFR) as N-containing precursor in the preparation of N-doped G/C-encapsulated Si nanoparticle/CNF composite (NG/C@Si/CNF) as anode material for LIBs. The preparation method used electrostatic attraction self-assembly, hydrothermal treatment, and calcining carbonization. In the prepared C-based composite material structure, NG/C@Si and CNF worked together to construct a stable and continuous conductive 3D structure. The structure provided an effective open channel for the migration of Li^+^ and good elastic buffer for the Si nanoparticles. It avoided the direct contact between the Si nanoparticles and the electrolyte. This work realized an LIBs anode material with high reversible capacity, high rate performance and high Li storage capacity compared to the previous results.

## Methods

### Materials and chemicals

Iron (III) nitrate nonahydrate (98%), copper (Cu) (II) nitrate trihydrate (99%), aluminum nitrate nonahydrate, molybdate tetrahydrate, ammonium carbonate, hydrogen peroxide (H_2_O_2_, 30%), melamine (≥ 99.0%), and citric acid anhydrous (≥ 99.5%) were purchased from Daejung Chemical and Metals Co (Gyeonggi-do, Korea). Formaldehyde (37 wt% in water), sulfuric acid (95–98%), and ethyl alcohol anhydrous (99.9%) were purchased from Sigma–Aldrich (St. Louis, USA). Si nanoparticles powder (APS ≤ 50 nm, 98%) was purchased from Alfa Aesar, Inc (MA, USA). GO (0.5%) was purchased from Angstron materials (Gyeonggi-do, Korea). APTES (≥ 99%) was provided by AcroSeal (United Kingdom. All reagents of analytical grade and materials were used as received. Deionized (DI) water was used in the preparation of aqueous solutions.

### Synthesis of CNF

The CNF was synthesized by a co-precipitation method to prepare Fe–Cu (70:30 at.%) bimetallic catalysts. The catalysts were synthesized by annealing, calcination, carbonization, and other processes by the chemical vapor deposition method (see Supplementary Information (SI) Fig. S1).

### Synthesis of Si-NH_2_

In order to modify the surface of silicon nanoparticles, we first dipped SiNPs into a piranha solution (sulfuric acid/hydrogen peroxide = 3:1 v/v) to introduce hydroxyl groups on the surface to form Si–OH. In a beaker containing 30 ml of sulfuric acid solution, and slowly drop 10 ml of hydrogen peroxide solution into it under stirring, so that the two are fully mixed to form a uniform piranha solution. Then, 0.3 g of SiNPs was added to the solution and stirring in a water bath at 80 °C for 8 h. The mixed solution was vacuum filtered and washed with DI water several times to remove excess piranha solution on the surface of Si nanoparticles. Then, the pretreated Si nanoparticles were dried at 60 °C for 24 h in an oven. Disperse the dried Si nanoparticles in 500 ml DI water, add 20 ml APTES, and continue stirring for 24 h. Finally, the resulting mixed solution was washed with DI water several times to remove excess APTES, and then dried at 60 °C for 24 h in an oven. It is called Si-NH_2_.

### Synthesis of NG/C@Si/CNF composites

Dissolve citric acid in 200 ml of DI water, then add 0.3 g of modified Si-NH_2_. Ultrasonicate and stir evenly, then add 0.3 g of prepared CNF. Disperse by ultrasonication for 2 h and continue stirring for 1 h. 60 ml of 0.5% GO solution was added to the suspension. Ultrasonicate for 2 h and then stir for 30 min, so that the various substances were mixed thoroughly through simple physical processes that yield a highly stable dispersion. Then, 30 ml of formaldehyde was added dropwise to the suspension of GO/Si/CNF under continuous stirring. After thoroughly mixing for 30 min, 6 g melamine (molar ratio of melamine/citric acid was 3:1) was added with stirring for 30 min. The resulting solution was transferred into a Teflon-lined stainless-steel autoclave and heated at 180 °C for 12 h. After cooling to room temperature, the products after the hydrothermal synthesis were vacuum filtered, washed with DI water, and dried in an oven at 60 °C for 24 h to obtain the GO/MFR@Si/CNF composites. The dried composites were heated to 800 °C at a rate of 10 °C/min in a quartz tube furnace with argon (Ar) gas flow and kept for 5 h to obtain the thermally reduced NG/C@Si/CNF composites.

**Figure 1:**
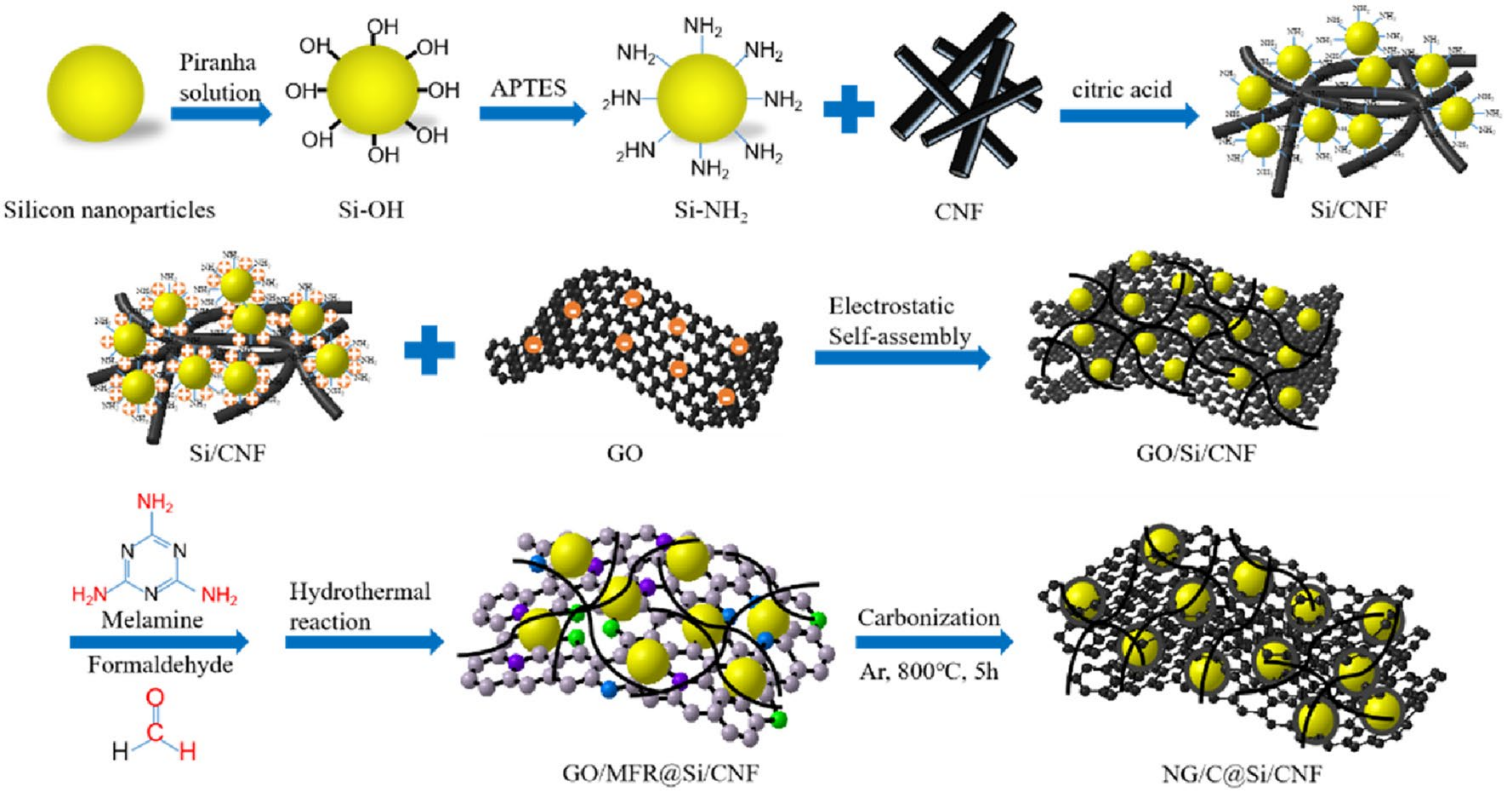
Schematic diagram for the preparation process of NG/C@Si/CNF composite material.

For the electrochemical performance comparisons, NG/C@Si and G@Si/CNF composites were also prepared by the above process, with the appropriate addition of the CNF and MFR, respectively. The mass ratio of Si, formaldehyde, citric acid, melamine, GO, and CNF used was consistent with that in the preparation of NG/C@Si/CNF composites. A schematic illustration of the preparation process is shown in Fig. 1.

### Materials characterization

The surface morphology and microstructure of the NG/C@Si/CNF, NG/C@Si, and G@Si/CNF composites were characterized by field-emission scanning electron microscopy (FE-SEM using a S-4800 from Hitachi (Tokyo, Japan) and high-resolution transmission electron microscopy (HRTEM) using a JEM-2100 from JEOL (Tokyo, Japan). Qualitative and quantitative analyses of the elements in the prepared samples were carried out by energy dispersive x-ray spectroscopy (EDS) mapping using an ARL-3460 from Thermo Fisher Scientific (MA, USA). The sample composition and crystal structure were examined by taking powder x-ray diffraction (XRD) measurements using an Ultima IV from Rigaku (Tokyo, Japan) with a 2 kW system and Cu-Kα radiation (K = 1.5418 Å). The scanning range was the 2θ range of 5°–90°. Raman spectroscopy was conducted on a LABRAM HR-800 from Horiba Jobin–Yvon (Paris, France) with a laser light (λ = 514 nm) in a wave number range of 100–3000 cm^−1^. Fourier transform infrared spectroscopy (FT-IR) using a 6700 spectrophotometer from Nicolet (WI, USA) was used to analyze the changes in the surface functional groups of a sample with potassium bromide pellet in the frequency range of 4000–500 cm^−1^. The chemical bonding states were determined by X-ray photoelectron spectroscopy (XPS) using a Multilab-2000 from Thermo Fisher Scientific(MA, USA) with a twin anode and Al-Kα radiation as the x-ray source. The amounts of each component in the composites were measured by thermo-gravimetric analysis (TGA) using a Diamond TG-DAT 8122 thermal analyzer system from Perkin Elmer (MA, USA). A sample was heated from 25 to 800 °C at a rate of 10 °C/min under an air atmosphere.

### Fabrication of LIBs and electrochemical measurements

Two-electrode batteries were prepared using NG/C@Si/CNF, NG/C@Si, and G@Si/CNF composites as anode active materials for LIBs. To test and characterize the electrochemical performances of the electrodes, the working electrodes were prepared by mixing active material, conductive agent (Super P), and binder (polyvinylidene fluoride) at a mass ratio of 8:1:1. The mixture was dissolved in an appropriate amount of N-methyl-pyrrolidinone with thinky mixer stirring to prepare a uniformly dispersed electrode active slurry. The resulting slurry was coated on a Cu foil current collector through a doctor-blade and dried at 120 °C for 24 h under vacuum to form an electrode plate. The electrodes were punched into a negative pole disk with a diameter of 12 mm. The measured average load density of each electrode was ~ 1.0 mg/cm^2^. Metallic Li foils (200 µm) as the counter and reference electrodes and two-electrode Li-ion coin cells (CR2032) were assembled in a high-purity Ar filled glove box. The separator membrane and electrolyte were polyethylene film (Tonen Chemical Corporation, 20 µm in thickness) and 1 M LiPF_6_ in a mixture of ethylene carbonate/ethyl methyl carbonate/diethyl carbonate (volume ratio of 1:1:1). The galvanostatic charge–discharge test and cycling performance of the cell were carried out using a battery testing system from Toscat-3000 System (Toyo, Japan) at a voltage range of 0.01–2.5 V (vs. Li/Li^+^). The specific capacity was calculated based on the whole mass of an anode material. Cyclic voltammetry (CV) was carried out at a scan rate of 0.1 mV s^−1^ in a voltage range of 0.01–2 V at room temperature using a VSP potentiostat analysis instrument from BioLogic. Electrochemical impedance spectroscopy (EIS) measurements were conducted using a VSP potentiostat electrochemical analysis instrument from BioLogic using a frequency range from 0.1 Hz to 1 MHz and amplitude of 5 mV.

## Results and discussion

### Structure and morphology

A schematic diagram of the preparation process for NG/C@Si/CNF composites is shown in Fig. 1. The MFR formed by polymerization of formaldehyde and melamine was used as N source and C shell precursor (see reaction process in SI Fig. S2). Citric acid was the cross-linking agent, while GO and CNF were the conductive matrix and C framework. The NG/C@Si/CNF composite was synthesized via electrostatic interactions. The dispersion of Si nanoparticles in piranha solution grafted the hydroxyl groups on the surface of Si nanoparticles to form Si–OH. The siloxane groups of APTES were easily grafted onto Si nanoparticles with –OH end caps and formed Si–NH_2_ through a hydrolysis process. The thickness of the modified layer of Si nanoparticles was controlled by the APTES hydrolysis time. The ionization of the carboxyl groups in GO and the amino group of Si-NH_2_ caused the formation of negative and positive charges, respectively. The electrostatic attraction between positive and negative charges occurred. The self-assembly reaction was hydrothermal. As the temperature was decreased gradually, MFR and citric acid aggregated on the surface of Si nanoparticles. The Si nanoparticles with –NH_2_ groups dispersed uniformly into the C-based precursor composed of MFR, citric acid, and CNF through electrostatic interactions. MFR and citric acid encapsulated the surface of Si nanoparticles and served as binding materials for the cross-linking Si nanoparticles, GO sheets, and CNFs. After high temperature calcination and carbonization, GO was reduced to G and MFR on the surface of Si nanoparticles and GO was converted to N-doped C and N-doped G, respectively. Due to the uniform dispersion of Si nanoparticles in the C-based precursor, Si nanoparticles were encapsulated uniformly in NG/C. The introduction of CNF also prevented Si nanoparticles from falling off from G.

**Figure 2:**
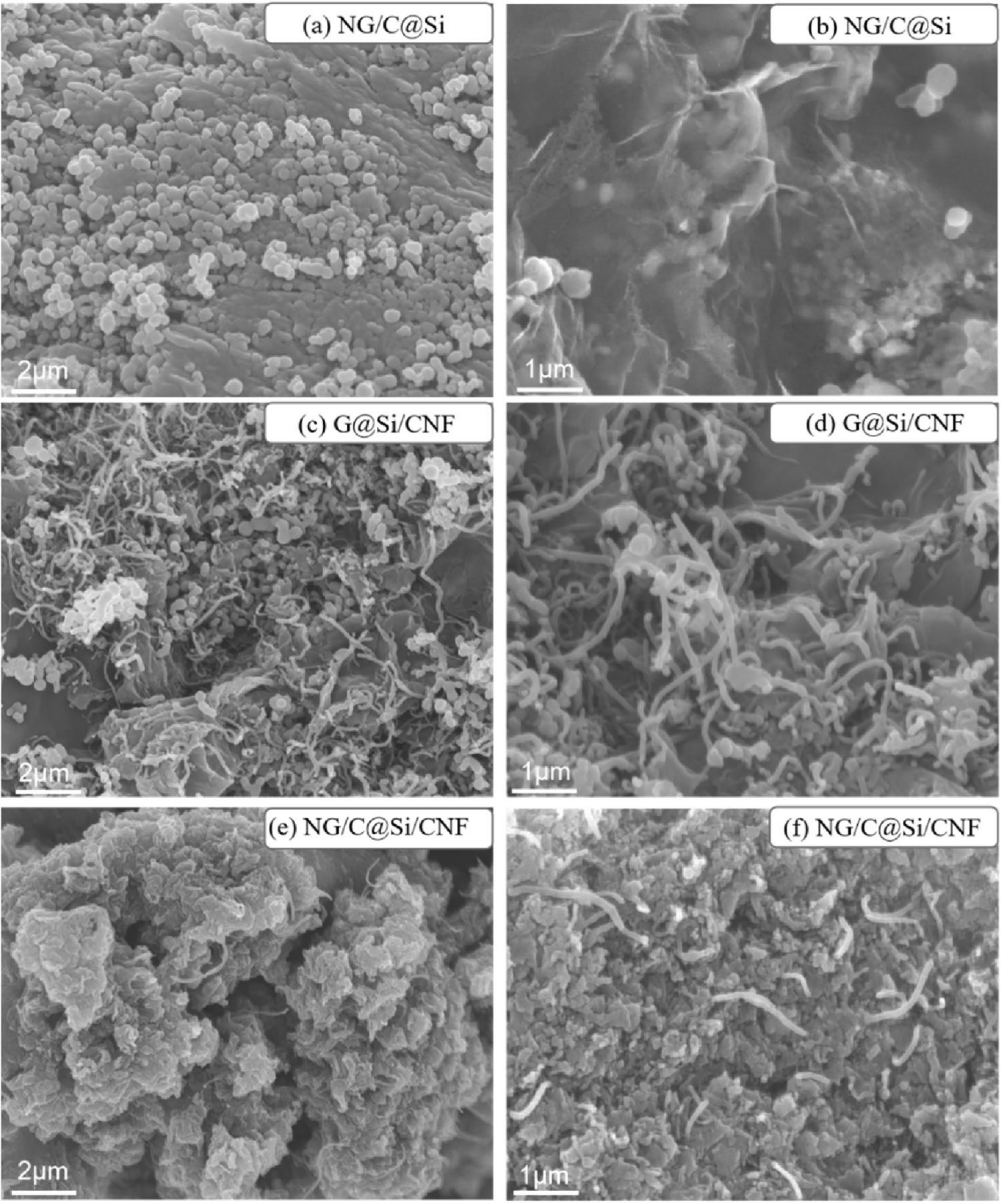
SEM images of the NG/C@Si (**a**, **b**), G@Si/CNF (**c**, **d**), and NG/C@Si/CNF (**e**, **f**) composites.

The morphologies and structures of NG/C@Si, G@Si/CNF, and NG/C@Si/CNF composites are shown in the representative SEM images in Fig. 2. The images of NG/C@Si composites without intercalated CNFs are shown in Fig. 2a and b. The uniformly dispersed Si nanoparticles were encapsulated in G without severe agglomeration. However, due to the volume change of the Si nanoparticles during charging and discharging, the particles were peeled off easily from G; thus, causing serious damage to the electrode structure. The images of G@Si/CNF composites without the introduction of MFR C matrix are shown in Fig. 2c and d. The Si nanoparticles were exposed and severe agglomeration of the G layer and the Si nanoparticles occurred. These caused the formation of a thick SEI layer on the surface of the Si nanoparticles. The SEI layer increased the resistance of the material and affected the transfer of electrons and Li^+^ inside the electrode; thereby, lowering the electrical conductivity of the composite material. The images of the NG/C@Si/CNF composite are shown in Fig. 2e and f. The Si nanoparticles were well wrapped by the C matrix composed of G and CNF. A few Si nanoparticles were exposed. The NG/C@Si encapsulated with Si nanoparticles was tightly wound by CNFs, forming a stable 3D structure. The structure showed good voids, which can accommodate the volume change of the electrode structure, reduce the pulverization of the electrode, and provide the electrode with enhanced mechanical integrity.

**Figure 3:**
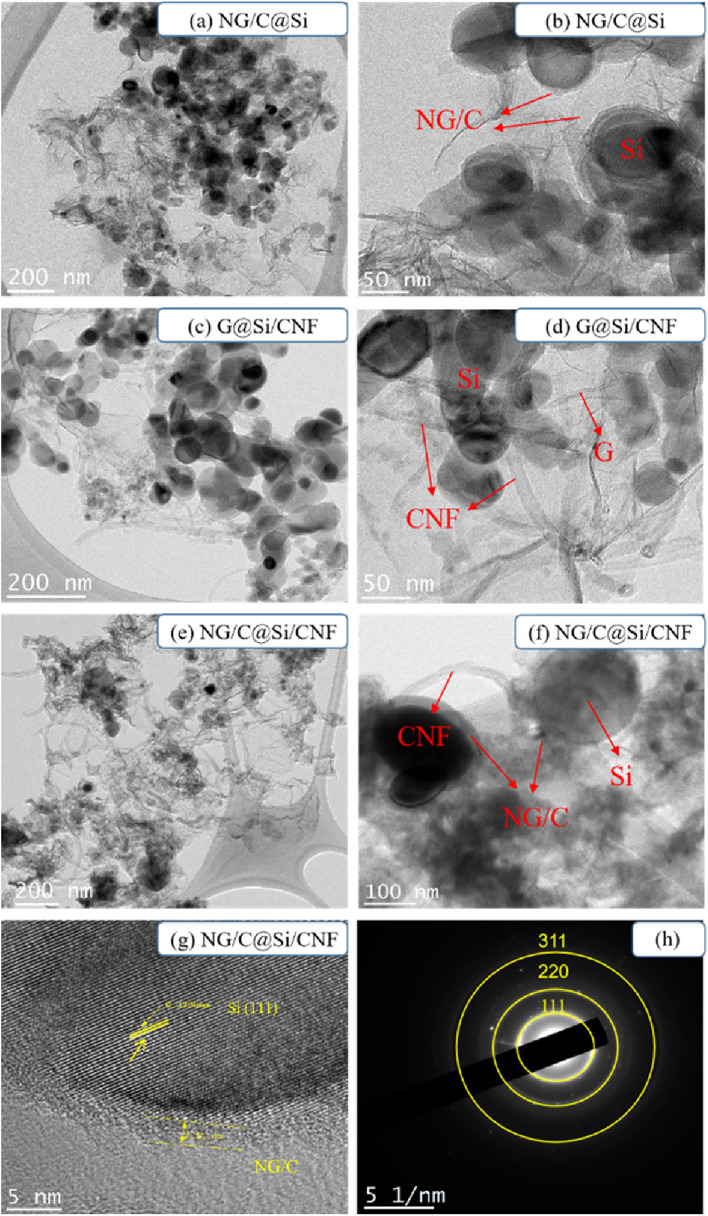
Representative TEM images of NG/C@Si (**a**, **b**), G@Si/CNF (**c**, **d**) and NG/C@Si/CNF (**e**, **f**) composites, (**g**) HRTEM images of NG/C@Si/CNF composites, and (**h**) SAED pattern of Si.

**Figure 4:**
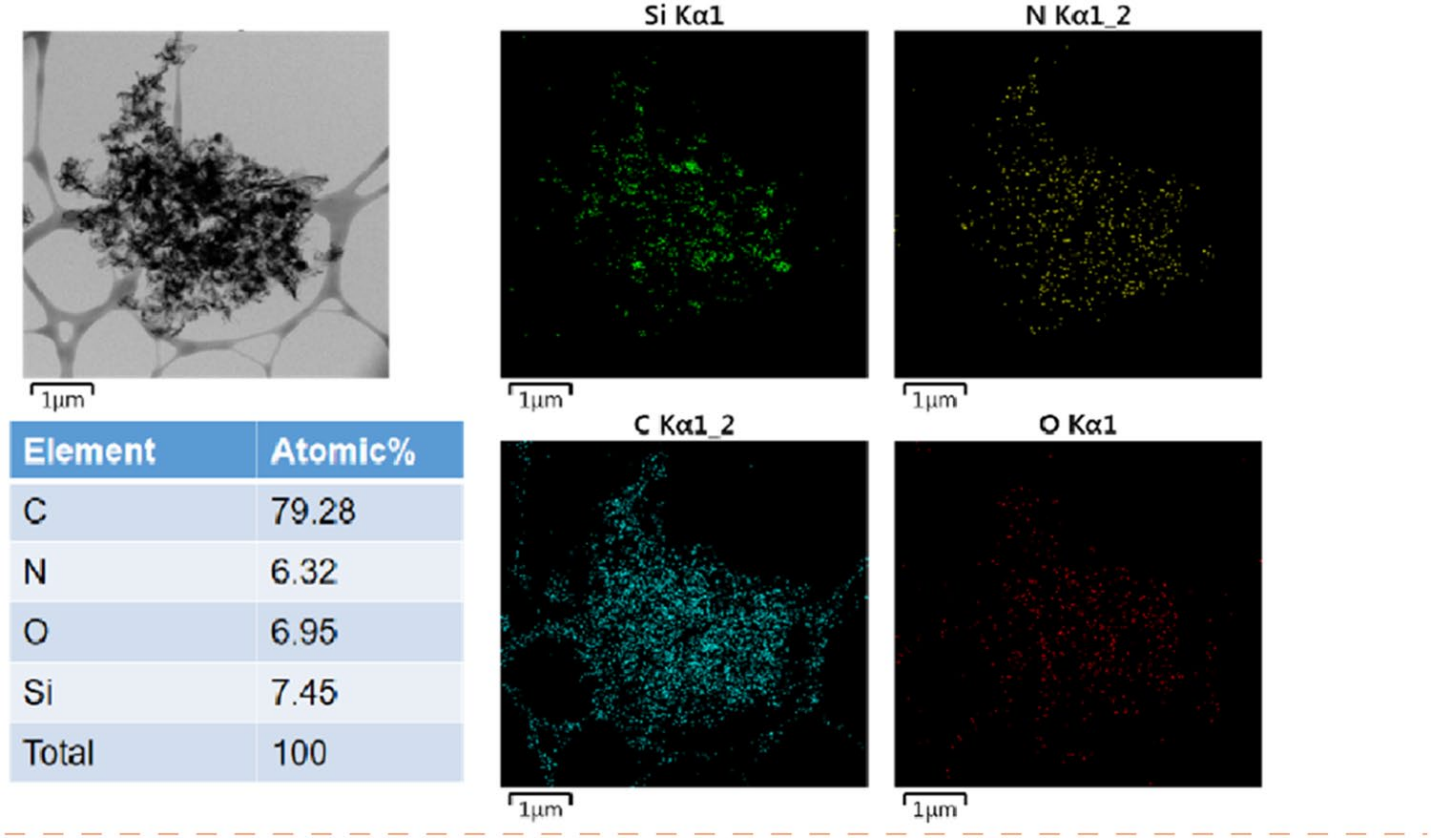
EDS mapping of the Si, N, C, and O elements on the surface of the NG/C@Si/CNF composite.

TEM image analysis was performed to magnify the structure and morphology of the composites (Fig. 3). Consistent with the SEM results, the NG/C@Si showed no CNF intercalation and the Si nanoparticles were uniformly dispersed on the wrinkled G sheets without severe agglomeration (Fig. 3a,b). The G@Si/CNF showed no introduction of MFR C matrix and the CNFs were interspersed between Si nanoparticles coated with wrinkled G to form a stable 3D structure (Fig. 3c,d). In the NG/C@Si/CNF composites (Fig. 3e,f), the Si nanoparticles were homogeneously embedded in G and the MFR layer coated on the surface of the Si nanoparticles well integrated in both CNF and NG/C@Si. The tight winding of the CNF prevented effectively the Si nanoparticles from falling off the G sheet and helped maintain the original electrode structure. The Si nanoparticles that were encapsulated in the C-based layer avoided the direct contact with the electrolyte, reduced the growth of the SEI layer, and reduced the resistance. The flexible C-based space accommodated and buffered the volume change of the Si nanoparticles. The interwoven CNFs in the complex provided efficient open channels for the transport of ions and electrons. Furthermore, the interplanar spacing of Si nanoparticles was 0.320 nm and the fringes were identified as Si(111) planes (Fig. 3g). The surface of the Si nanoparticles was coated with a distinct NG/C C layer. The bright diffraction spots in the three diffraction rings of a selected area electron diffraction (SAED) corresponded to the (111), (220), and (311) crystal planes of Si nanoparticles (Fig. 3h). The EDS elemental mapping image of the NG/C@Si/CNF composite is shown in Fig. 4. In addition to the Si element, we detected a uniform distribution of C, N and O elements in the sample, indicating that the N atoms were successfully doped into rGO and both MFR C-based layer and CNF were tightly bound to the modified Si nanoparticles.

**Figure 5:**
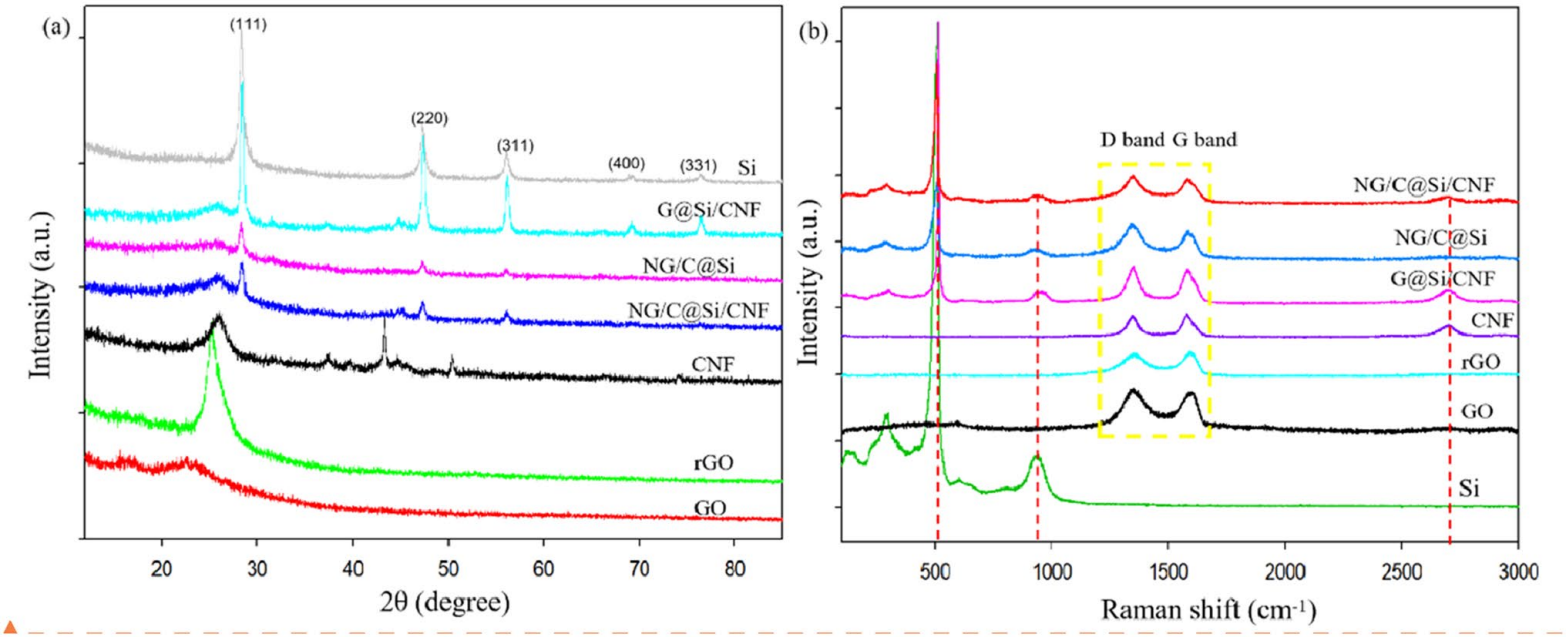
Representative (**a**) XRD patterns and (**b**) Raman spectra of Si, GO, rGO, CNF, NG/C@Si/CNF, NG/C@Si, and G@Si/CNF.

The XRD curves and Raman scattering spectra of Si, GO, rGO, CNF, NG/C@Si composite, G@Si/CNF composite, and NG/C@Si/CNF composite are shown in Fig. 5. The prepared composites of NG/C@Si, G@Si/CNF, and NG/C@Si/CNF showed peaks at 28.4°, 47.3°, 56.1°, 69.1° and 76.4°, which correspond exactly to (111), (220), (311), (400), and (331) planes of crystalline Si (JCPDS 27-1402)^31–33^. Thus, the crystal structures of the Si particles were unchanged during the preparation of the composite material. In the XRD curves of rGO and CNF, the broad diffraction peak at ~ 26° corresponds to the characteristic peak of amorphous C (002)^30,34^. These diffraction peaks were all present in the composites, indicating that rGO and CNF were distributed uniformly in each composite. In addition, the minute amount of C originating from the MFR after heat treatment may lead to broadening of the diffraction peaks. We note that the peak intensity of Si in the G@Si/CNF composite was strong. Meanwhile, the amorphous C in this composite is weaker than the NG/C@Si and NG/C@Si/CNF composites. This effect was attributed to the C-based 3D structure, which is composed of G and MFR in the NG/C@Si and NG/C@Si/CNF composites resulting in the uniform and extensive encapsulation of SiNPs^11^. However, the low content of CNF caused the absence of other the diffraction peaks of CNF in the composites. This was due to the relatively low intensity of the diffraction peaks compared with Si. In addition, compared with the GO XRD curve, the characteristic peak of GO in the rGO curve after high temperature calcination and reduction disappeared, A characteristic peak appeared at ~ 25°, which indicated the successful reduction of GO to rGO.

To further investigate the microstructure of amorphous C, Raman spectroscopy was performed and the spectra of all Si-based composites are compared in Fig. 5b. In the spectra of Si, G@Si/CNF, NG/C@Si, and NG/C@Si/CNF, the band located at 515 cm^−1^ corresponds to the typical Raman vibrational mode of crystalline Si, indicating that the Si was unchanged and has good crystallinity after forming the composite. For GO, rGO, CNF, G@Si/CNF, NG/C@Si, and NG/C@Si/CNF composites, two main peaks appeared at approximately 1348 and 1580 cm^−1^. These are derived from D-bands and G-bands, which are related to lattice defects of sp^3^-hybridized disordered C atoms and sp^2^-hybridized ordered C-type structures, respectively. The relative intensity ratio (ID/IG) of the D-band and G-band indicated the degree of graphitization, defect density, and graphitized domain size. The ID/IG values summarized in SI Table S1 are directly related to the defects and conductivity^13,14,31^. The ID/IG value of the NG/C@Si composite (1.03) was higher than G@Si/CNF composite (1.01), which indicated that the defect density was higher after N-doped. These defects provides more channels for the conduction of electrons and ions; thus, conducting better electricity. However, they also show that the degree of N-doped will lead to a decrease in the degree of graphitization of C materials. The ID/IG value of the NG/C@Si/CNF composite (1.02) is slightly smaller than NG/C@Si composite (1.03) and the degree of graphitization of the Si/C based composite material was improved. Therefore, the NG/C@Si/CNF composites improved both the N-doped degree and graphitization degree of the material.

**Figure 6:**
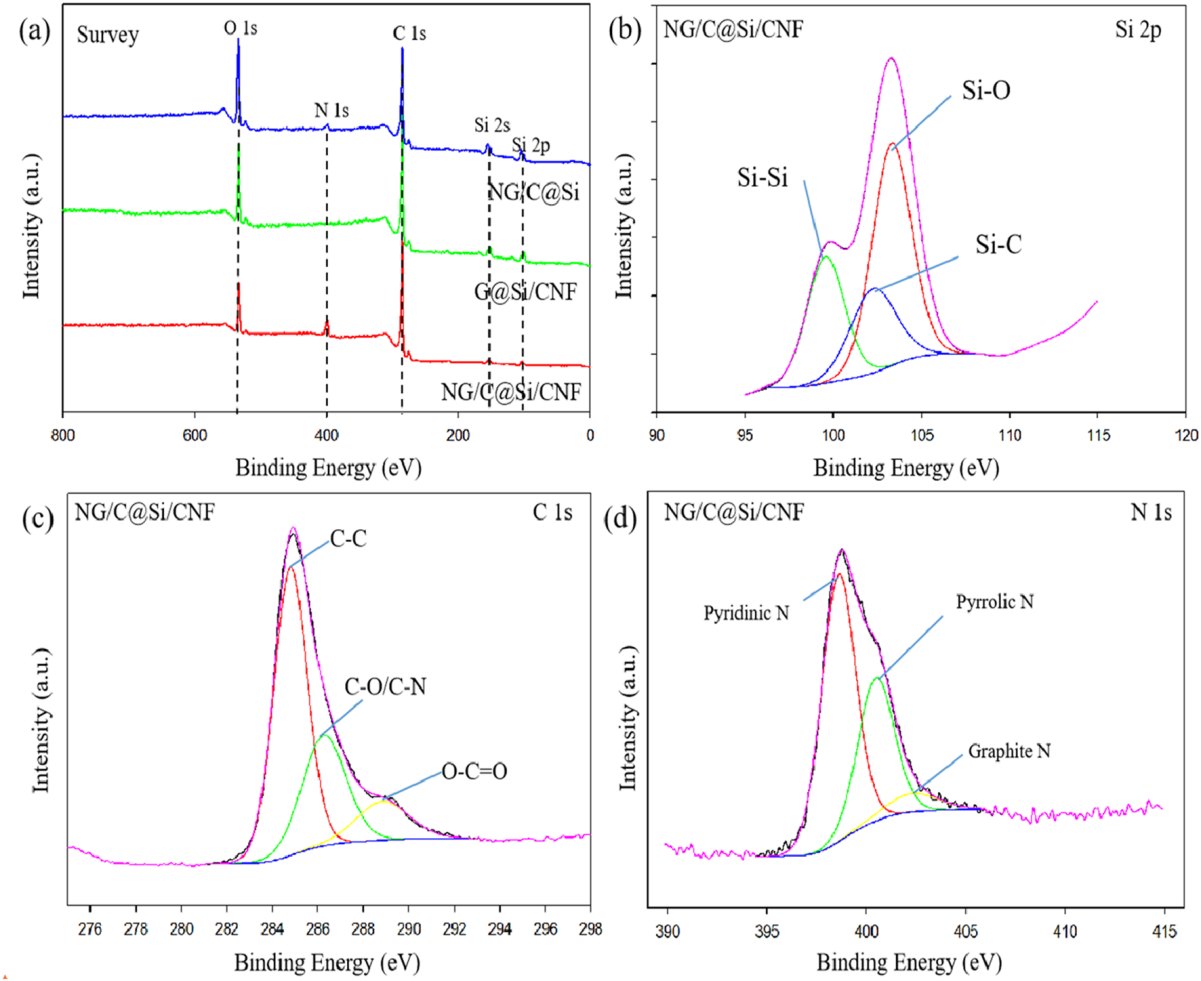
(**a**) Survey XPS spectra of NG/C@Si/CNF, G@Si/CNF, and NG/C@Si. (**b**) Si 2p XPS spectrum of NG/C@Si/CNF, (**c**) C 1*s* XPS spectrum of NG/C@Si/CNF, (**d**) N 1 s XPS spectrum of NG/C@Si/CNF.

XPS is an effective method to determine the relative surface elemental composition and chemical properties of nanocomposites. The XPS spectra of the materials are shown in Fig. 6. The elements Si, C and O coexisted in the three composites (Fig. 6a). However, the N element was only present in the NG/C@Si and NG/C@Si/CNF composites, indicating that this element was added successfully in these composites. The MFR layer underwent a deep carbonization process; thus, the relative peak of C atoms in the NG/C@Si/CNF composite was higher than the G@Si/CNF composite. The high carbonization level is beneficial in improving the electrical conductivity of composites^35,36^. The high-resolution Si 2p spectrum of the NG/C@Si/CNF composite is shown in Fig. 6b. The binding energy peaks at 99.57, 102.26 and 103.32 eV were attributed to Si–Si, Si–C and Si–O, respectively. The existence of Si–O was due to the high surface activity of Si nanoparticles and the slight oxidation that occurred on the surface of the particles during the experiment. The Si–O thin layer enabled the dispersion on Si nanoparticles in water and reduced the agglomeration of Si nanoparticles in the sample^37^. The Si–C bond is an indicative of the formation of a carbon shell on the surface of the Si particles. It is indicated that after high temperature carbonization, the strong interaction between Si and carbon makes the carbon layer closely adhere to the surface of Si particles and tightly encapsulate the Si particles. The Si 2p spectra of Si–NH_2_ and Si–OH are shown in SI Figs. S3a and S3b, respectively. An increase in the Si–O peak (103.2 eV) was observed, which was attributed to the APTES with many Si–O bonds and the surface of Si nanoparticles that were covered by APTES. The weakening of the XPS signal of Si–Si bonds was due to the limited probe depth of XPS. Furthermore, compared with the Si 2*p* spectrum of Si-NH2, the peaks of Si–O in the Si 2*p* spectrum of GO/MFR@Si/CNF (SI Fig. S3c) and NG/C@Si/CNF (SI Fig. S3d) were increased significantly. This was a result of the GO introduction. The Si–O peak in the Si 2*p* spectrum of NG/C@Si/CNF was weaker than GO/MFR@Si/CNF, which was the result of thermal reduction. In C 1*s* (Fig. 6c), the three peaks observed at 284.5, 286.3 and 288.5 eV were the C–C, C–O/C–N and O–C=O bonds, respectively. For the N 1*s* spectrum (Fig. 6d), the three peaks at 398.5, 400.9 and 403.2 eV are the characteristic peaks of pyridine N, pyrrolic N and graphitic N, respectively. The appearance of C-N in the C 1*s* and N 1*s* spectra suggests the presence of N element in the C matrix due to the doping process. Pyridine N and pyrrolic N are N atoms that were substituted for C atoms at the edge or defect site of G, resulting in a large number of vacancies for storage of Li-ions. This is useful in improving the Li storage capacity of C materials. Graphite N that is formed by replacing internal C atoms with N atoms to form C–N covalent bonds is beneficial for improving the electrical conductivity of G sheets^31,38,39^.

**Figure 7:**
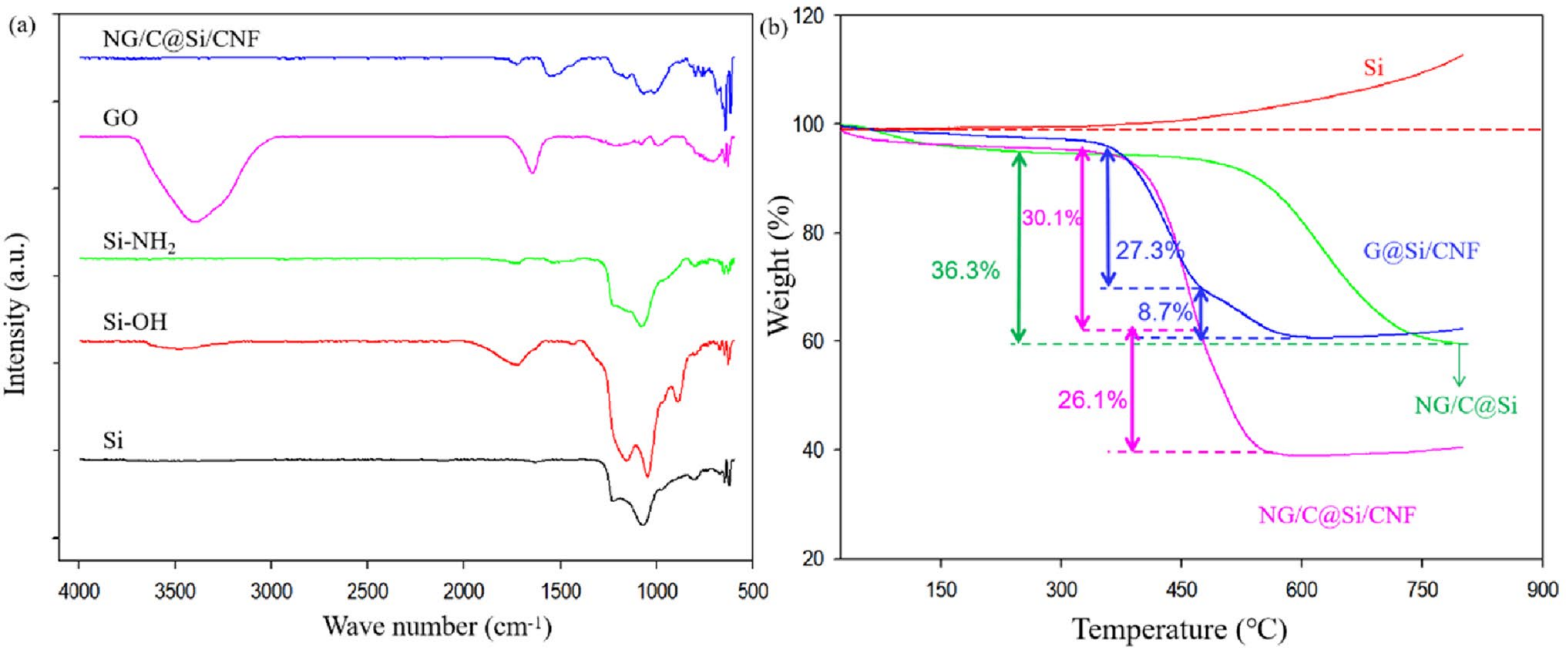
(**a**) FT-IR spectra of pure nano-Si, Si–OH, Si–NH_2_, GO, and NG/C@Si/CNF. (**b**) TGA curves of pure nano-Si, G@Si/CNF, NG/C@Si, and NG/C@Si/CNF composite materials.

The surface chemical structures of the relevant samples were determined by FT-IR (Fig. 7a). The Si, Si–OH, and Si–NH_2_ all showed strong absorption peaks in the range of 1200–1000 cm^−1^, which corresponded to the asymmetric stretching and bending of siloxane groups (Si–O–Si). The Si–O–Si groups on the surface of pure Si nanoparticles were due likely to the formation of a small amount of SiO_2_ layer due to air oxidation. This was verified by the Si 2*p* XPS spectrum. Next, the Si, Si–OH, and Si-NH_2_ samples showed an absorption peak in the range of 1800–1600 cm^−1^, which corresponded to the vibration of water molecules. The absorption peak of the Si–OH sample was strongest, which indicated that more water was adsorbed on the surface of the sample due to the presence of hydrophilic –OH groups. The Si–OH sample also showed an obvious absorption peak at around 3500 cm^−1^, which was attributed to the vibration of the –OH group. This indicating that the –OH group was grafted successfully on the surface of the Si nanoparticles^30^. In the FT-IR spectrum of the GO sample, the absorption peak at 1639 cm^−1^ was due to the stretching vibration of C=C. The broad band at 3380 cm^−1^ was due to the strong stretching vibration of the -OH group. The absorption peaks at 1719, 1186 and 1075 cm^−1^ were due to the stretching vibration of the –C=O bond in –COOH, C–OH and C–O bonds, respectively. In the NG/C@Si/CNF sample spectrum, the absorption peak at the 3380 cm^−1^ was not found, indicating that the –OH group was removed after thermal reduction. In addition, the two new absorption peaks at 1559 and 1227 cm^−1^ was due to the stretching vibrations of C=N and C–N, respectively, indicating the successful N doping^40^.

The TGA curves of pure Si nanoparticles, NG/C@Si, G@Si/CNF, and NG/C@Si/CNF in the temperature range of 25–800 °C are shown in Fig. 7b. Based on the results, the weight of pure Si nanoparticles and other composites increased after 600 °C. This was due to the difficulty in oxidizing the interior of Si nanoparticles and forming SiO_2_ on the surfaces. These are consistent with the XPS results. The weights of the G@Si/CNF and NG/C@Si/CNF composite samples dropped rapidly between 430 and 460 °C. This was due to the first decomposition of CNF. The weight of the NG/C@Si and NG/C@Si/CNF samples dropped rapidly at 560 °C, which was mainly due to the degradation of rGO and non-oriented C coatings and rapid oxidation. The calculated C contents in NG/C@Si, G@Si/CNF, and NG/C@Si/CNF were 36.3%, 36.0% and 56.2%, respectively. The content in the NG/C@Si/CNF samples was significantly higher than the other samples, which indicated the successful carbonization of MFR after high temperature calcination. The calculated contents of silicon nanoparticles in NG/C@Si, G@Si/CNF and NG/C@Si/CNF are 63.7%, 64% and 43.8%, respectively. At the beginning of the experiment, the mass ratio of Si nanoparticles and carbon nanomaterials was 1:1 when we prepared the composites. However, the carbon content in the NG/C@Si/CNF composite is higher than that of Si nanoparticles. This is due to the carbonization of the MFR layer when the composite is calcined at high temperature, and the high temperature leads to the loss of a small amount of Si nanoparticles.

### Electrochemical performance

**Figure 8:**
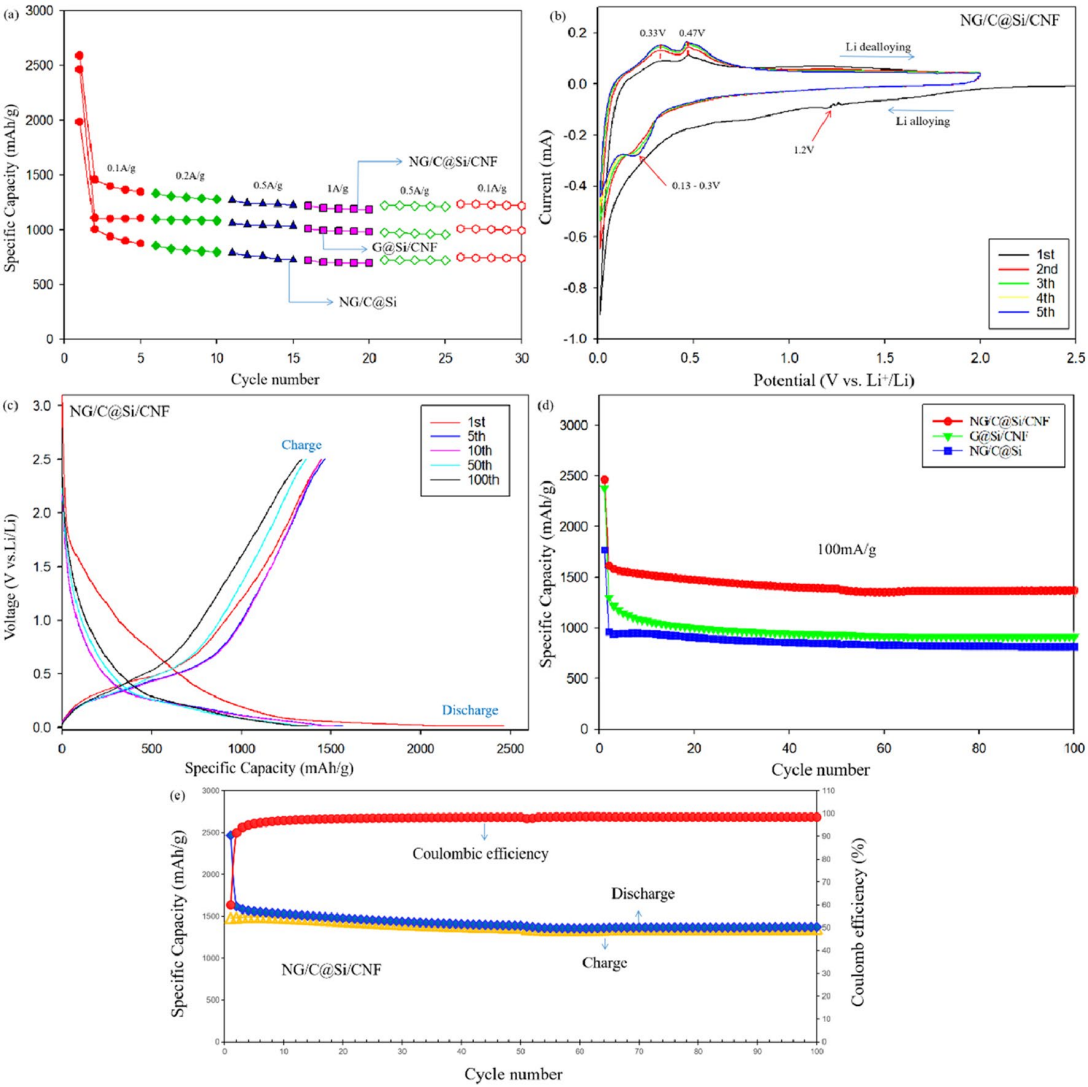
(**a**) Rate performances of the G@Si/CNF, NG/C@Si, and NG/C@Si/CNF electrodes under different current densities. (**b**) CV curves of the NG/C@Si/CNF electrode in the initial five cycles. (**c**) The charge and discharge profiles of the NG/C@Si/CNF composite electrode. (**d**) Cycling performances of the G@Si/CNF, NG/C@Si, and NG/C@Si/CNF composite electrodes at a current density of 0.1 A g^−1^. (**e**) Charging and discharging cycle performance and Coulombic efficiency of the NG/C@Si/CNF composite electrode at a current density of 0.1 A g^−1^.

The rate capability values of G@Si/CNF, NG/C@Si, and NG/C@Si/CNF electrodes at various current densities in the range of 0.1–1 A/g are summarized in Fig. 8a. The discharge specific capacity values gradually decreased with the increase in the current density values. The NG/C@Si/CNF electrodes exhibited better cycling performance and rate capability compared with the G@Si/CNF and NG/C@Si. The specific capacities of NG/C@Si/CNF electrodes were 1346.20, 1278.18, 1224.89 and 1185.23 mAh/g at current densities of 0.1, 0.2, 0.5 and 1 A/g, respectively. When the current density recovered to 0.1 A/g, the discharge specific capacity recovered to 1218.61 mAh/g. The excellent cycling performance and rate capability of the NG/C@Si/CNF composites was attributed to the several reasons. First, the N atoms in the graphitic planes formed C–N covalent bonds in the G sheets, which changed the electron density of C. The substitution of N atoms for C atoms at the edges increased the vacancies and defects, enhancing the electrochemical Li-ion storage ability of the composites. Therefore, the G and C (from pyrolyzed MFR) coating facilitated the conduction of electrons. Second, the embedded CNFs enhanced the electron transport paths that appeared inside the composites. As more electrons were transported through the C framework, more Si nanoparticles in the composite were activated. Finally, the supporting role of the amorphous C layer buffered the sharp volume change of Si nanoparticles during a charge/discharge event. The addition of CNF also formed a strong 3D structure, which accommodated effectively the volume change and prevented the electrode structure from cracking. This also prevented the Si particles from falling off the C matrix due to the enlarged surface area; thus, effectively maintaining the structure of the entire electrode.

The CV curves of the coin cells, which were fabricated from NG/C@Si/CNF composite electrodes during the first four cycles, within the potential voltage range of 0.01–1.5 V (vs. Li^+^/Li) at scan rate of 0.1 mV s^−1^ are shown in Fig. 8b. The CV curve of the first cycle was different from the subsequent cycles, especially during the discharge. In the cathodic scan of the first cycle, a broad peak appeared at ~ 1.2 V, which may be due to the reaction between electrode material and electrolyte and the formation of an irreversible SEI on the electrode surface. The cathodic peak disappeared in the next cycle, indicating the formation of a stable SEI film on the electrode surface. This was consistent with the charge/discharge test results and resulted in an irreversible loss of the initial capacity. The reduction peak at 0.13–0.3 V was attributed to the amorphous Li_x_Si alloy, which formed from the interaction between amorphous Si and Li^+^. The two broad oxidation peaks at 0.33 and 0.47 V corresponded to the delithiation decomposition of Li_x_Si alloy to amorphous Si. The intensities of all anodic and cathodic peaks increased with the number of scans. This was due to the gradual activation of the composite electrode material during cycling and consistent with previous reports on Si-based materials. The CV scan curves of G@Si/CNF and NG/C@Si electrode are shown in SI Figs. S4a and S5a, respectively. In the cathodic scan, cathodic and reduction peaks that were related to the SEI film and alloy formation, respectively also appeared. However, in the CV curve of NG/C@Si, the curve in the first cycle was not stable and a weak cathodic peak also appeared in the subsequent cycles. These suggested that the SEI film formed on the surface of the NG/C@Si electrode was more unstable than the other two electrodes. This instability affects significantly the electrochemical performance of the electrode material.

The charge and discharge profiles (1st, 5th, 10th, 50th and 100th cycles) of the NG/C@Si/CNF composite electrode at a current density and voltage range of 0.1 A/g and 0.01–2.5 V are shown in Fig. 8c. The electrode exhibited a long and flat discharge profile at around 0.1 V in the 1st cycle, which corresponded to the formation of amorphous Li_x_Si alloys. At this cycle, the discharge capacity, charging capacity, and initial coulombic efficiency values were 2464.8 mAh/g, 1477.7 mAh/g and 60%, respectively. The high specific capacity during the 1st discharge suggested that the electrodes were fully lithiated at low current densities. The loss of irreversible capacity at the initial cycle was due to the SEI film formed, the contact between the surface of the active material and the electrolyte, and the oxygen-containing functional groups on the surface of the active materials, which underwent irreversible side reactions with Li^+^^37,41^. After the 1st cycle, the discharge curves overlapped, which indicated a stable electrode. The charge and discharge profiles of the G@Si/CNF and the NG/C@Si composite electrodes are shown in SI Figs. S4b and S5b, respectively. For the G@Si/CNF composite, the discharge capacity, charging capacity, and initial coulombic efficiency values were 2381.6 mAh/g, 1145.1 mAh/g and 48.1%, respectively. For the NG/C@Si composite, the discharge capacity, charging capacity, and initial coulombic efficiency values were 1768.6 mAh/g, 896.1 mAh/g and 50.1%, respectively. For the NG/C@Si/CNF composite, the volume change of Si was suppressed by the encapsulation of Si nanoparticles in the C base layer. The increase in the degree of graphitization of the active material and the reduction of oxygen-containing functional groups on the surface of the material after high temperature calcination also improved the coulombic efficiency^42^.

Further comparison of the electrochemical performance of the composites was conducted. The cycling performance of the NG/C@Si/CNF, G@Si/CNF, and NG/C@Si composites at a current density of 0.1 A/g are shown Fig. 8d. For the G@Si/CNF composite (SI Fig. S4c), the capacity was maintained at 912.7 mAh/g after 100 cycles with coulombic efficiency of 97.8%. The initial discharge capacity of the NG/C@Si composite was 1768.6 mAh/g, which decayed to 868.3 mAh/g after 100 cycles as shown in SI Fig. S5c. The coulombic efficiency was 97.8%. This was likely caused by the volume change of Si particles after several lithiation/delithiation processes, which cracked the Si particle surface and exfoliated the Si particles from the G sheets; thus, reducing the electrochemical performance of the composites. The severe aggregation of Si particles may also lead to the loss of electrochemical performance. Compared with the G@Si/CNF and NG/C@Si composite electrodes, the NG/C@Si/CNF composite electrode exhibited a much better cycle performance. The initial discharge capacity of the NG/C@Si/CNF composite electrode was 2464.8 mAh/g. After 100 cycles, the discharge capacity remained at 1371.4 mAh/g with capacity retention rate of 55.6%. Meanwhile, the coulombic efficiency reached 98.4% as shown in Fig. 8e. The electrochemical test results are summarized in SI Table S2. In addition, we also provide the cycling performance of the NG/C@Si/CNF samples under high loading state (about 2 mg/cm^2^), as shown in Fig. S6. The good cycling performance of the NG/C@Si/CNF composite electrode was attributed to the 3D cross-linked structure formed by the close contact among CNF, C layer, and Si nanoparticles. This structure maintained the stability and integrity of the electrode. The introduction of CNF and the protection of G and C layers on the surface of Si nanoparticles buffered the volume change and prevented the exfoliation of Si nanoparticles from the G surface due to the volume change during cycling. The direct exposure of the Si nanoparticles to the electrolyte was prevented and a stable SEI film was formed. After surface modification, the bonding between the C layer and the Si nanoparticles was strengthened through electrostatic attraction, which improved the structure stability. With the introduction of CNF, more reactive sites were added in N-doped G due to the existence of many vacancies and defects. The uniform distribution of the modified Si nanoparticles also reduced the aggregation and accumulation of particles. These provided more efficient channels for the conduction of ions and electrons and promoted the transfer of ions and electrons that enhanced the conductivity of the electrode materials.

**Figure 9:**
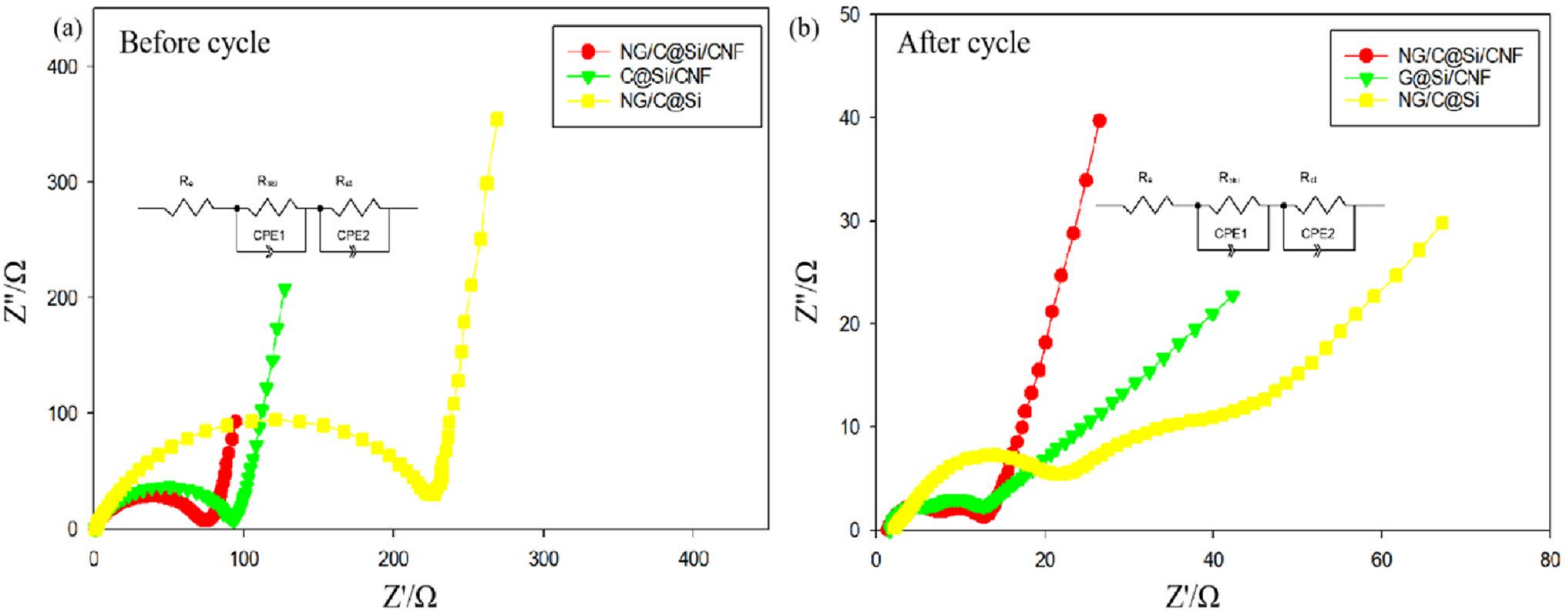
Nyquist plots and electrochemical impedance spectra of the G@Si/CNF, NG/C@Si, and NG/C@Si/CNF composite electrodes (**a**) before and (**b**) after the cycle.

To elucidate the chemical reaction kinetics, the EIS patterns of the different electrodes were studied at the frequency range from 1 mHZ to 0.1 HZ at an amplitude ratio of 5 mV. The results are shown in Fig. 9. An equivalent circuit for fitting impedance was inserted in Fig. 9, where R_e_, R_SEI_, and R_CT_ represent the resistances of ion transport in the electrolyte solution, Li^+^ migration through the surface membrane, and charge transfer, respectively. CPE1 and CPE2 are the surface film and double-layer capacitors, respectively. In the Nyquist plots, the curves appeared as semicircles in the middle to high frequency regions and slanted lines in the low frequency region. The diameter of the semicircle is related to the resistance of Li-ions through the insulating layer on the surface of the active material particles (R_SEI_) and the charge transfer resistance (R_CT_). The slanted line corresponds to the diffusion resistance of Li-ions within the electrode active material. The diffusion resistance can be expressed by the Warburg impedance (Z_W_). The Nyquist plots obtained for NG/C@Si/CNF, G@Si/CNF, and NG/C@Si electrodes with R_CT_ values of 74.7, 92.8 and 227.0 Ω, respectively, before cycling are shown in Fig. 9a. Compared to the other electrodes, the semicircle diameter was the smallest and the diagonal line was the shortest compared in the NG/C@Si/CNF electrode. These were due to the 3D conductive network composed of C coatings, CNFs, and Si nanoparticles, which provided faster charge transport channels, effectively facilitated electron transfer, reduced charge transfer resistance, and improved electrochemical performance. To verify the formation of a stable SEI film, the Nyquist plots of the NG/C@Si/CNF, G@Si/CNF, and NG/C@Si electrodes after 100 cycles were investigated. The results are shown in Fig. 9b. The R_SEI_ values were 5.2, 6.8 and 22.5 Ω, respectively, which suggested that the MFR-based C coating alleviated the volume expansion of Si nanoparticles and helped in the formation of a stable SEI film. The SEI film formed on the NG/C@Si/CNF electrode during a long period was the thinnest; thus, the resistance value was the lowest. The low resistance was attributed to the activation of the composite electrode and the complete contact among the electrolyte, electrode material, C layer, and rGO. These provided a stable buffer layer and enhanced the electronic and ionic conductivity during cycling. Bode plots can be used to assess the effectiveness of lithium-ion diffusion in the electrode materials. It is reported that the electrode with a smaller phase angle in the low-frequency region of the Bode plot shows better capacitive performance and faster lithium-ion diffusion^43^. There is no significant difference in the Bode plots of NG/C@Si/CNF, G@Si/CNF, and NG/C@Si electrodes before the cycle, as shown in Fig. S7a. However, the phase angle of NG/C@Si/CNF electrode is much smaller than that of the other electrodes in the low-frequency region after 100 cycles, as shown in Fig. S7b. This indicates that lithium-ions diffuse faster in NG/C@Si/CNF electrode than G@Si/CNF and NG/C@Si electrodes. This is because the 3D structure of NG/C@Si/CNF electrode accommodated the volume change of Si and maintained the integrity of the electrode structure during cycles, which leads to the decent cycling performance of NG/C@Si/CNF electrode, as explained in the previous section(Fig. 8).

**Figure 10:**
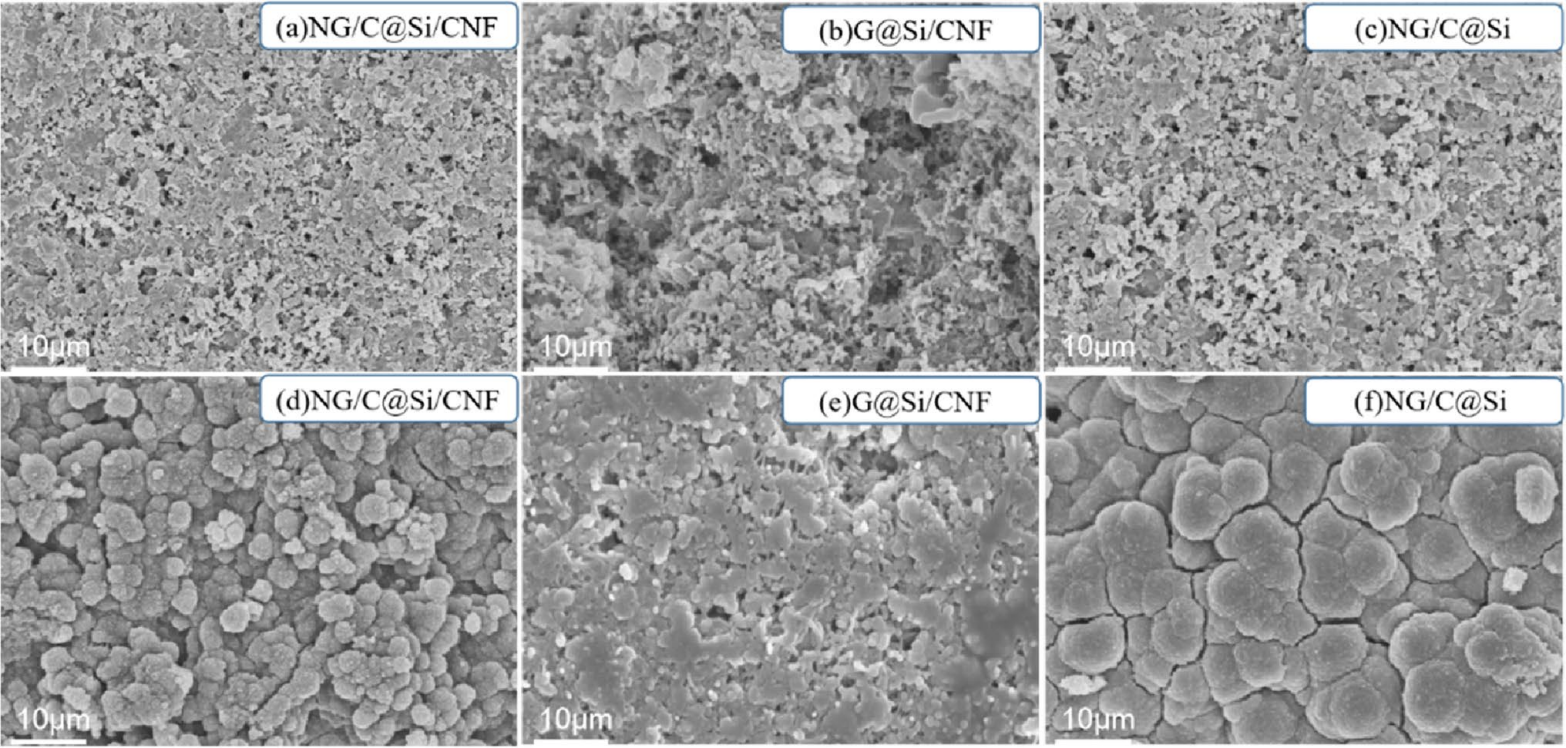
Representative SEM images from surface view of NG/C@Si/CNF, G@Si/CNF and NG/C@Si electrodes (**a**, **b**, **c**) before the first cycle and (**d**, **e**, **f**) after 100 lithiation/delithiation cycles at a current density of 0.1 A g^−1^.

To further explore the Li storage mechanism of different composites, we compared the SEM images of the NG/C@Si/CNF, G@Si/CNF, and NG/C@Si composite electrodes (see Fig. 10). We focused on the surface topographic features before cycling and after 100 cycles in the lithiated state. After cycling, the original Si particles, G, and CNF were not clearly observed on the surfaces of the electrodes. This was due to the side reactions, which occurred during a cycle. The products of these reactions accumulated on the electrode surface and covered the active materials (e.g., Si particles). After 100 cycles, the surface of the NG/C@Si composite electrode showed more cracks compared with the other electrodes. These cracks would lead to poor contact among the active materials. The shrinkage and expansion of the Si volume during the repeated lithiation/delithiation process cause the Si nanoparticles to be easily pulverized and peeled off from the surface of a C material. Thus, the integrity of the electrode is affected seriously, resulting in a rapid capacity decay. In the cross-section of an electrode material shown in SI Fig. S8, the thickness of the NG/C@Si/CNF electrode after lithiation expanded from 34.6 to 38.6 µm after lithiation. The thickness of the G@Si/CNF electrode before and after cycling expanded from 3.2 µm (before cycling) to 8.8 µm (after cycling), and the thickness of NG/C@Si electrode was significantly expanded from 36.7 µm (before cycling) to 44.2 µm (after cycling). Compared with the NG/C@Si/CNF electrode, the other two electrodes suffered from severe volume expansion. However, the volume of the NG/C@Si/CNF electrode was not severely affected after lithiation and its particle volume expansion was small. This was because of the 3D structure formed by the interaction of Si nanoparticles, C layer, and CNF, which accommodated the volume change of Si and maintained the integrity of the electrode structure. This stronger evidence further demonstrated the superior electrochemical performance of the NG/C@Si/CNF electrode. Moreover, after lithiation, we observed obvious cracks between the active material and the Cu foil in the G@Si/CNF and NG/C@Si electrodes. The cracks were a result of severe volume changes in the electrodes. Separation of the current collector from the active material typically results in a loss of electrode electrochemical performance. In addition, the surfaces of the G@Si/CNF and NG/C@Si electrodes showed thick and non-uniform SEI films after lithiation. The thick SEI film will increase the resistance and affect the cycling performance of the electrode material.

## Conclusion

In this study, we successfully encapsulated Si nanoparticles in N-doped C layers, which were composed of G and MFR base layers through electrostatic interactions with carboxyl and amino groups, hydrothermal reactions, and carbonization. The CNF and NG/C layers improved the conductivity of the electrode material and buffered effectively the volume change of the Si material during charging and discharging. In addition, the Si nanoparticles encapsulated by the C layer reduced effectively the direct contact with the electrolyte; thus, forming a stable SEI film. The CNF linked the Si nanoparticles that were wrapped with the C layer, preventing the cracking and exfoliation of the Si nanoparticles; thus, maintaining the integrity of the electrode structure. N-doped introduced successfully pyridine N, pyrrolic N, and graphitic N into G, resulting in more vacancies and defects in the G structure. This provided more efficient transport channels for the intercalation of Li-ions. In addition, the surface modification of Si nanoparticles improved the dispersibility in solution and enhanced the binding between the N-doped C layer and the Si nanoparticles. This produced uniformly distributed Si nanoparticles in the system and reduced the agglomeration of G and Si nanoparticles. The highly stable 3D network structure composed of Si nanoparticles, CNFs, and C layers shortened the transport distance of ions and facilitated the transfer of ions and electrons, which improved the conductivity of the electrodes. Notably, at a current density of 100 mA/g, the NG/C@Si/CNF electrode maintained a high reversible specific capacity of 1371.4 mAh/g after 100 cycles. The prepared electrodes exhibited excellent cycle stability and rate performance, which could serve as reference in the development of anode materials for Li-ion batteries.

## Supplementary Information

Supplementary Information.

## Data Availability

The datasets generated during and/or analyzed during the current study are available from the corresponding author on reasonable request.
