# Supplementary material for: Three-dimensional network of nitrogen-doped carbon matrix-encapsulated Si nanoparticles/carbon nanofibers hybrids for lithium-ion battery anodes with excellent capability
Source: Sci Rep. 2022 Sep 26;12:16002. doi: 10.1038/s41598-022-20026-9 (PMC9512820; doi:10.1038/s41598-022-20026-9)
Supplement: Supplementary file 1 — Supplementary Information. [file 41598_2022_20026_MOESM1_ESM.docx]

**Supplementary Information (SI)**

Three-dimensional Network of Nitrogen-doped Carbon Matrix-encapsulated Si Nanoparticles/Carbon Nanofibers Hybrids for Lithium Ion Battery Anodes with Excellent Capability

Ruye Cong^a^, Minsang Jo^b^, Angelica Martino^a^, Hyun-Ho Park^a^, Hochun Lee^b^,

Chang-Seop Lee^a,*^

^a^Department of Chemistry, Keimyung University, Daegu 42601, Korea

^b^Department of Energy Science and Engineering, DGIST, Daegu 42988, Korea

*Corresponding author: Chang-Seop Lee

Department of Chemistry, Keimyung University, Daegu 42601, Korea

Phone No: +82-53-580-5192; Fax No: +82-53-580-5056

E-mail: [surfkm@kmu.ac.kr](mailto:surfkm@kmu.ac.kr)


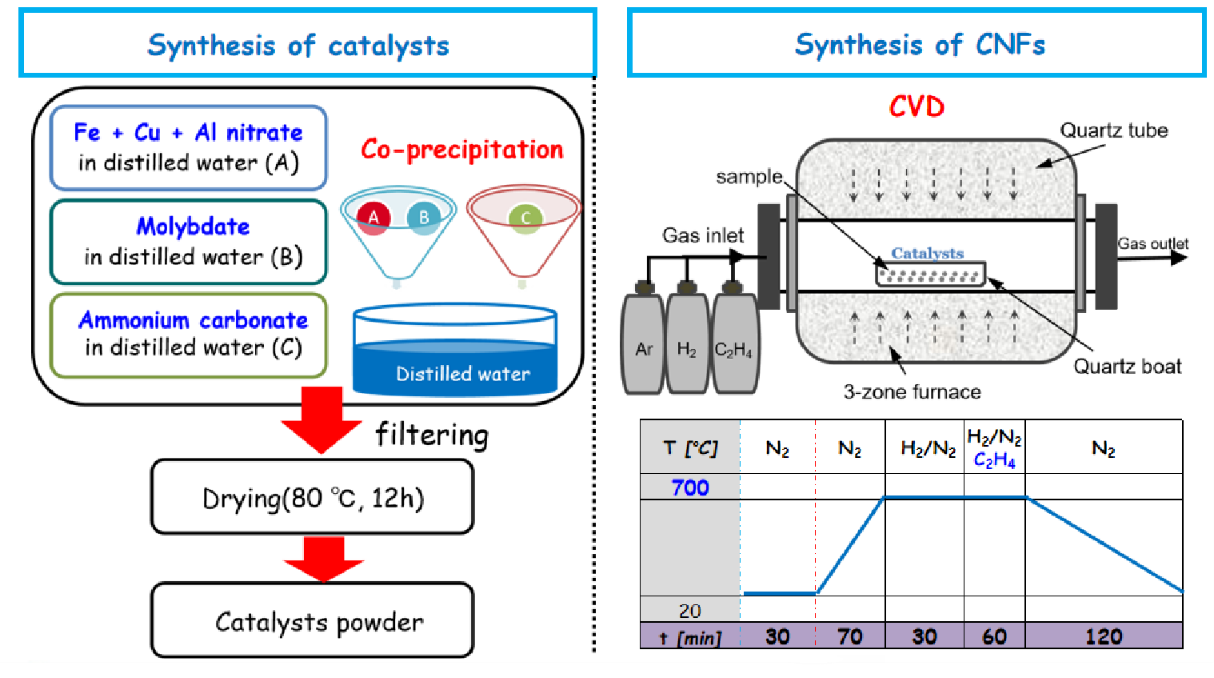


**Fig. S1.** Proposed process for preparing the catalysts and synthesis of carbon nanofibers (CNFs).


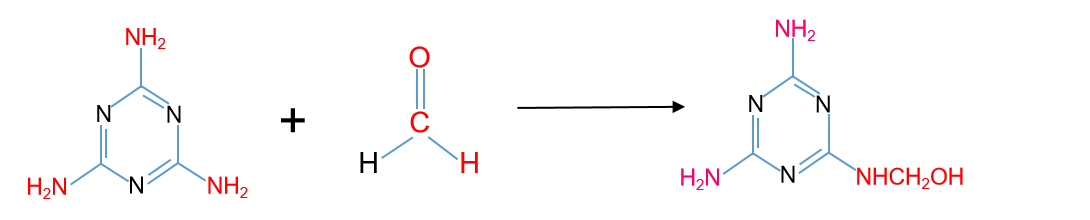


**Fig. S2.** Schematic diagram of the chemical reaction process of the melamine formaldehyde resin (MFR) formed by the polymerization of formaldehyde and melamine.


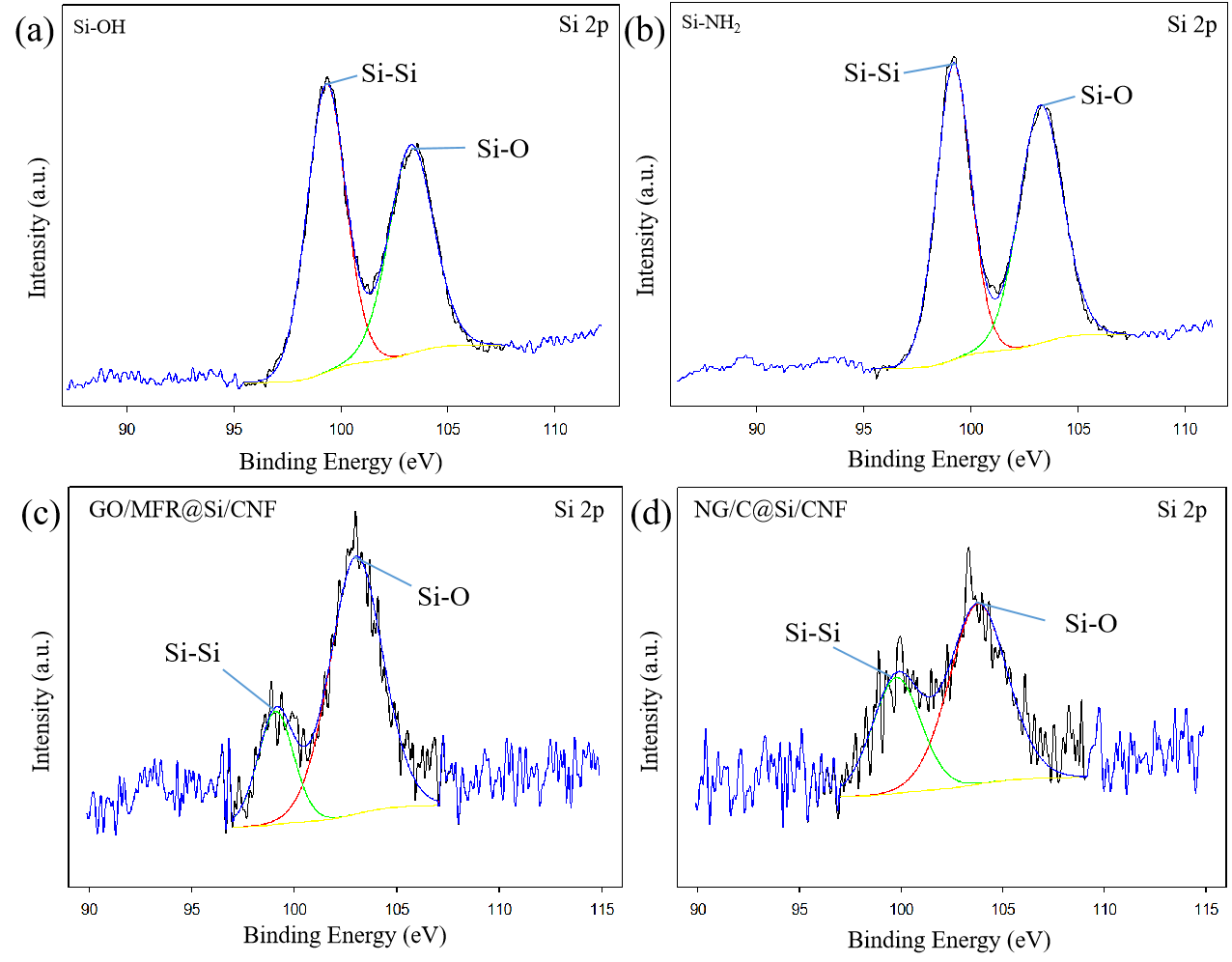


**Fig. S3.** Representative Si 2p XPS spectra of (a) Si-OH, (b) Si-NH_2_, (c) GO/MFR@Si/CNF, and (d) NG/C@Si/CNF.


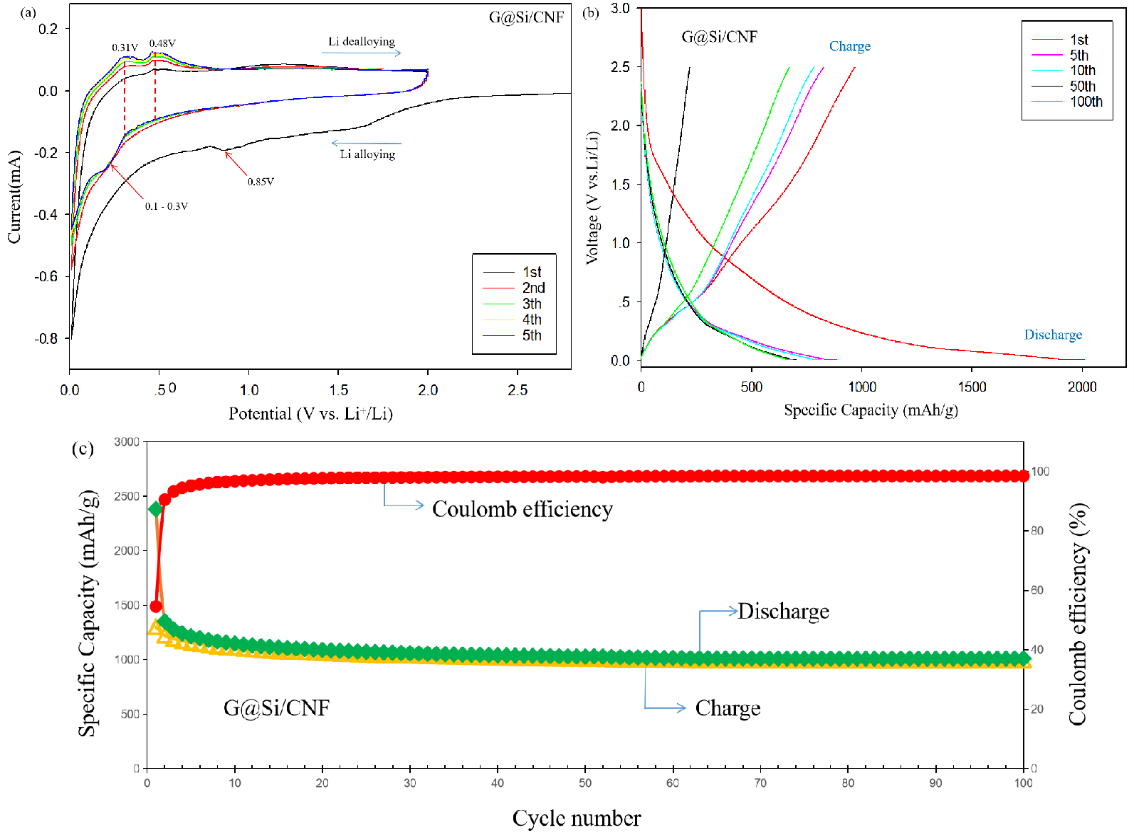


**Fig. S4.** (a) Cyclic voltammetry curves of the G@Si/CNF composite electrode in the initial five cycles. (b) The charge and discharge profiles of G@Si/CNF composite electrode. (c) The cycling performances of the synthesized G@Si/CNF composite electrode at a current density of 0.1 A·g^−1^.


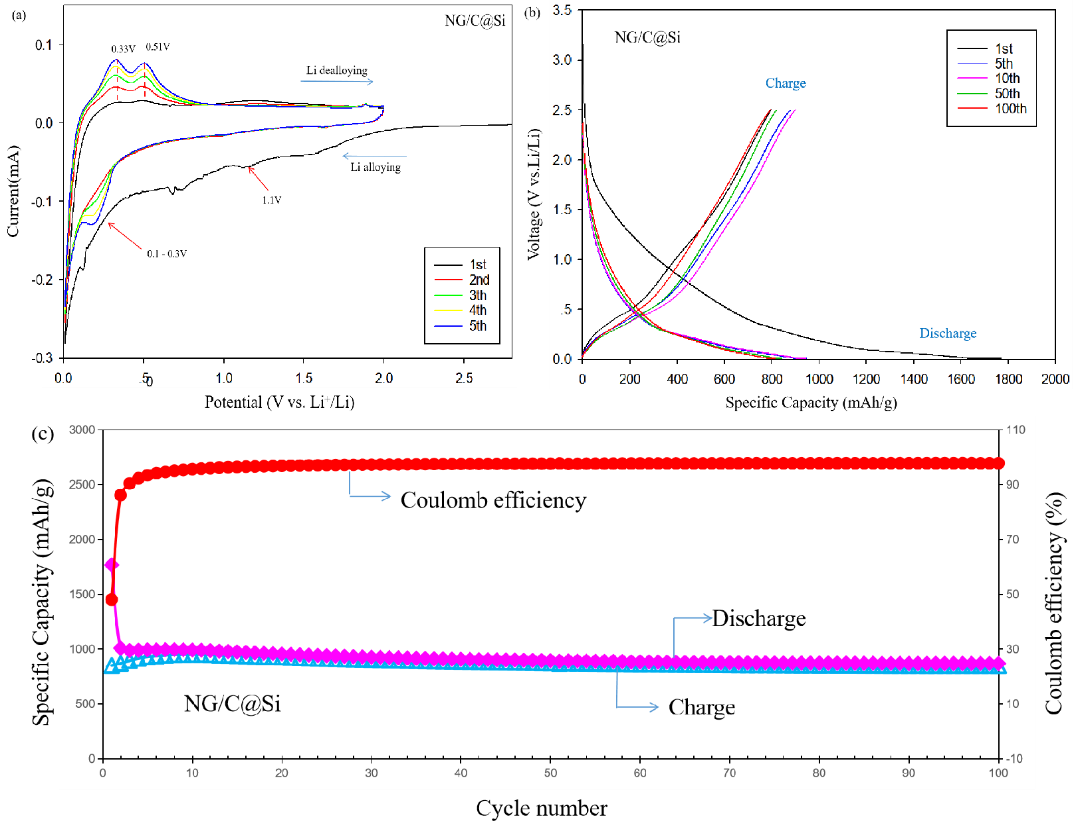


**Fig. S5.** (a) Cyclic voltammetry curves of the NG/C@Si composite electrode in the initial five cycles. (b) The charge and discharge profiles of NG/C@Si composite electrode. (c) The cycling performances of the synthesized NG/C@Si composite electrode at a current density of 0.1 A·g^−1^.


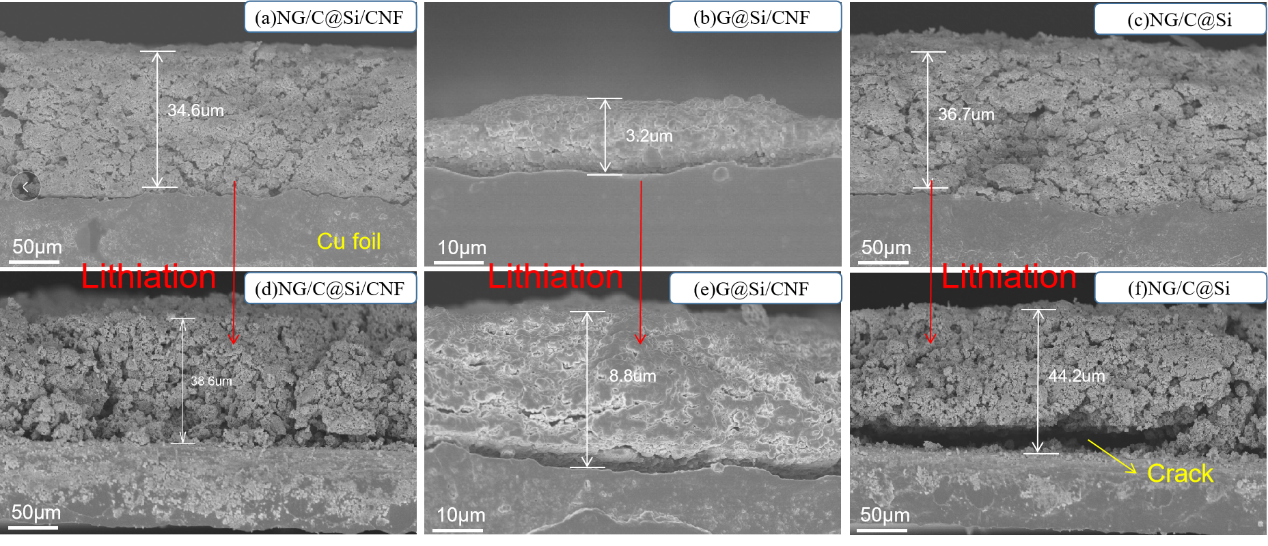


**Fig. S6.** Representative SEM images from cross section view of the NG/C@Si/CNF, G@Si/CNF, and NG/C@Si electrodes (a, b, c) before the first cycle and (d, e, f) after 100 lithiation/delithiation cycles at a current density of 0.1 Ag^−1^.

**Table S1.** Raman spectroscopy results for the GO, rGO, CNFs, NG/C@Si/CNF, G@Si/CNF, and NG/C@Si samples (see Fig. 5b).

| Sample | ID/IG |
| --- | --- |
| GO | 1.05 |
| rGO | 0.99 |
| CNF | 0.99 |
| G@Si/CNF | 1.01 |
| NG/C@Si | 1.03 |
| NG/C@Si/CNF | 1.02 |

**Table S2.** Discharge capacity, Coulomb efficiency, and capacity retention rate of the NG/C@Si/CNF, G@Si/CNF, and NG/C@Si composite electrodes.

| Samples | Max. discharge  capacity  (mAh/g) | Discharge capacity (mAh/g) | | Coulomb efficiency (%) | | Capacity retention rate (%) | |
| --- | --- | --- | --- | --- | --- | --- | --- |
|  |  | **After 50 cycle** | **After 100 cycle** | **first cycle** | **100 cycle** | **After 50 cycle** | **After 100 cycle** |
| NG/C@Si/CNF | 2464.8 | 1388.4 | 1371.4 | 59.9 | 98.4 | 56.3 | 55.6 |
| G@Si/CNF | 2381.6 | 1027.8 | 1010.3 | 54.6 | 98.5 | 47.6 | 46.9 |
| NG/C@Si | 1768.6 | 894.7 | 868.3 | 48.1 | 97.8 | 50.5 | 49.1 |
